# On the Relevance of Natural Stimuli for the Study of Brainstem Correlates: The Example of Consonance Perception

**DOI:** 10.1371/journal.pone.0145439

**Published:** 2015-12-31

**Authors:** Marion Cousineau, Gavin M. Bidelman, Isabelle Peretz, Alexandre Lehmann

**Affiliations:** 1 International Laboratory for Brain, Music and Sound Research (BRAMS), Montreal, QC, Canada; 2 Center for Research on Brain, Language and Music (CRBLM), Montreal, QC, Canada; 3 Department of Psychology, University of Montreal, Montreal, QC, Canada; 4 Institute for Intelligent Systems, University of Memphis, Memphis, TN, United States of America; 5 School of Communication Sciences & Disorders, University of Memphis, Memphis, TN, United States of America; 6 Department of Otolaryngology Head & Neck Surgery, McGill University, Montreal, QC, Canada; Virginia Commonwealth University, UNITED STATES

## Abstract

Some combinations of musical tones sound pleasing to Western listeners, and are termed consonant, while others sound discordant, and are termed dissonant. The perceptual phenomenon of consonance has been traced to the acoustic property of harmonicity. It has been repeatedly shown that neural correlates of consonance can be found as early as the auditory brainstem as reflected in the harmonicity of the scalp-recorded frequency-following response (FFR). “Neural Pitch Salience” (NPS) measured from FFRs—essentially a time-domain equivalent of the classic pattern recognition models of pitch—has been found to correlate with behavioral judgments of consonance for synthetic stimuli. Following the idea that the auditory system has evolved to process behaviorally relevant natural sounds, and in order to test the generalizability of this finding made with synthetic tones, we recorded FFRs for consonant and dissonant intervals composed of synthetic and natural stimuli. We found that NPS correlated with behavioral judgments of consonance and dissonance for synthetic but not for naturalistic sounds. These results suggest that while some form of harmonicity can be computed from the auditory brainstem response, the general percept of consonance and dissonance is not captured by this measure. It might either be represented in the brainstem in a different code (such as place code) or arise at higher levels of the auditory pathway. Our findings further illustrate the importance of using natural sounds, as a complementary tool to fully-controlled synthetic sounds, when probing auditory perception.

## Introduction

In Western music, the concept of consonance refers to a harmony, a chord, or an interval that is considered musically stable. The counterpart of consonance is dissonance and refers to unstable combinations of tones or chords that are used to create tension in music. Basic aspects of consonance and dissonance perception have been widely investigated through the use of isolated combinations of two or three simultaneously sounding tones (dyads or triads). Consonant intervals are consistently rated as pleasant, whereas dissonant intervals are consistently rated as unpleasant e.g. [1,2–4].

The acoustic correlates of consonance and dissonance have been widely debated over the past century. Helmholtz’s [5] roughness theory states that the unpleasantness of dissonant chords is due to amplitude modulation or “beating” produced by the interaction of harmonics too closely spaced to be resolved by the cochlea, that translate to an unpleasant feeling of roughness in dissonant chords. Stumpf’s [6] tonal fusion theory, on the other hand, rests on the observation that the component frequencies of consonant chords combine to produce an aggregate spectrum that is typically harmonic—where frequencies of the individual partials are multiples of a common fundamental frequency–and that this explains the pleasantness of consonant chords. Dissonance could thus be perceptually unpleasant either because the resulting spectrum of dissonant chords is inharmonic or because the concurrent presentation of tones produces beating or roughness. These competing theories have been hard to disentangle because inharmonicity and beating often co-occur in natural stimuli and the two theories share common qualitative predictions. However, recent work suggests that the harmonicity of the spectrum (as originally proposed by Stumpf) is the acoustic correlate of consonance and that roughness is rather heard by listeners as a pleasantness dimension that is orthogonal to consonance [4,7].

However, the question of how harmonicity is extracted by the auditory brain and at what level neural correlates of consonance-dissonance emerge remains unclear. Over the past few years, there has been increasing research on possible subcortical correlates of musical consonance, starting with Bidelman and Krishnan [8] who introduced, inspired by work on neural correlates of pitch by Cariani and Delgutte [9, 10], a new measure to quantify neural harmonicity called Neural Pitch Salience (NPS). NPS is a measure based on the assumption that the auditory system performs an autocorrelation analysis to compute pitch-related information [11–13]. The analysis features temporally based autocorrelation and harmonic pitch sieve analyses. This periodic sieve analysis is essentially a time-domain equivalent to the classic pattern recognition models of pitch in which a “central pitch processor’ matches harmonic information contained in the stimulus to an internal template in order to compute the heard pitch [14].

The scalp-recorded brainstem frequency-following response (FFR) is a sustained “neurophonic” potential with putative generators in the rostral brainstem [15,16] which reflects spectrotemporal properties of the eliciting acoustic stimulus. Previous studies demonstrated that NPS measured from FFRs correlates with behavioral reports of consonance and dissonance [8,17–19]. With the initial idea of maximizing FFR responses, these studies used synthetic tones with specific (but artificial) spectra (complex tones with 5–6 equal amplitude partials of a harmonics series). However, behaviorally relevant stimuli have been shown to yield the largest [20], or the most informative [21,22] neural responses in a number of species. Following the idea that our brain is not only optimized for natural sounds but also that using natural sounds to probe the auditory system is the best way to understand the neural computations that underlie our comprehension of speech and music [23], we extended the study of brainstem correlates of consonance and dissonance to natural sounds. We asked whether neural harmonicity (as measured via NPS) could capture consonance and dissonance across a wide variety of sound timbres that evoke these common percepts in humans. This question is especially important considering the growing number of studies that use this measure as an established brainstem correlate of consonance or to study a variety of other processes.

## Material and Methods

### Participants

Fourteen “Western listeners” participants (7 males, mean age 26.6 ± 4.1 years) provided written informed consent; all reported normal hearing and no history of hearing disorder or neurological disease. Data about music education was not collected. The experimental procedures conformed to the World Medical Association’s Declaration of Helsinki and were approved by the Research Ethics Committee of the Faculty for Arts and Sciences of the University of Montreal.

### Stimuli

Three musical dyadic (i.e., two-tone) intervals were constructed by combining two single tones from each of three instrumental categories. The three intervals—Unison (Un), dissonant Minor 2nd (m2) and consonant Perfect 5th (P5)—were chosen because they generated the biggest dynamic range in terms of neural and behavioral responses in previous studies [8,24]. For every dyad, the lower of the two pitches was fixed at G#3 (F0 = 207.65 Hz). Dyads were presented dichotically and the lowest pitch was always presented to the left ear. Tones were either natural recordings from the equal tempered scale: a saxophone (Sax) or a trained female vocalist (Voice), or synthesized complex tones containing the first six harmonics of the harmonic series with equal amplitude (sCT). Synthetic complex tones lasting 200ms were generated for the purpose of the study. For natural sounds, a 200ms segment was extracted from the steady-state portion of the stimuli used previously (McDermott et al., 2010). A 10ms square-cosine ramp was applied to all stimuli. A total of nine stimuli were constructed, representing three musical intervals (Unison, m2 P5) from the three different sound timbre categories (Sax, Voice, sCT).

### FFR Recording protocol

Participants reclined comfortably in an acoustically and electrically shielded booth. They were instructed to relax and avoid moving. FFRs were recorded from each participant in response to 3000 repetitions of each of the nine different stimuli at an intensity of 78 dBA through magnetically shielded insert earphones (Etymotic ER-2). Subjects watched a silent, subtitled movie. Stimuli were presented in randomized order with a stimulus-onset asynchrony of 290 ms. The experimental protocol intended to reproduce Bidelman and Krishnan [8] as closely as possible. Stimulus presentation was dichotic (one note per ear). Dichotic presentation was used to minimize peripheral contributions to consonance (e.g., cochlear beating/roughness) and isolate responses from a “central pitch processing mechanism” [25,26]. Stimuli were presented with a custom Matlab program (The Mathworks), interfaced with a signal processing system (RX6, Tucker-Davis Technologies).

Auditory-evoked brainstem potentials were recorded from five sintered Ag/AgCl electrodes that contained pre-amplification within the electrode housing (“active electrodes”, BioSemi). Ultra-flat active electrodes were placed on the midline of the forehead at the hairline (Fz), on the right mastoid (M1) and on the seventh cervical vertebra (C7). Two ground electrodes were placed on the central forehead. Active electrodes provide impedance transformation on the electrode to prevent interference currents from generating significant impedance-dependent nuisance voltages. We therefore did not control electrode impedances, but rather kept direct-current offset close to zero during electrode placement. Electrode signals were amplified with an ActiveTwo amplifier (BioSemi) with a dedicated bank of hardware amplifiers for low-noise brainstem recordings. The signals were sampled at 16384 Hz and stored for offline analysis using ActiView software (BioSemi).

Adequate measures were taken to minimize the occurrence of stimulus artifacts (electromagnetically shielded booth, shielded transducers), and visual inspection of individual FFRs did not reveal any stimulus artifact. Additionally, control recordings in which the exact same procedure was followed but with the inserts sitting outside rather than inside the ear resulted in no signal above the noise floor. This, together with the observation that very little energy was observed above 1kHz (i.e., above the limit of phase-locking) confirmed that all FFRs were of neural origin and did not contain electromagnetic stimulus artifact [27,28].

### FFR Data analysis

The data was processed using the EEGLAB toolbox [29] and in-house scripts developed in Matlab. EEGs were bandpass-filtered offline between 80 and 3000 Hz (Butterworth, 12dB/octave). EEG recordings at electrode Fz were referenced to C7 (vertebrae) and then segmented into 309-ms epochs ranging from −20 to 289 ms relative to the stimulus onset. Epochs containing unusually large potentials (> ±50 μV) were rejected prior to averaging. Further epochs were rejected using EEGLAB’s automatic iterative rejection procedure with an initial threshold of five standard deviations [29]. Epochs for each stimulus type were averaged to obtain nine FFR waveforms per subject.

We measured the neural pitch salience (NPS) of each brainstem response using the methods described by Bidelman & Krishnan (2009). First, we computed the autocorrelation function (ACF) of each FFR time waveforms to index the periodicities contained in each brainstem response. Each ACF was then weighted with a decaying exponential (*τ* = 20 ms) to give greater precedence to shorter pitch intervals [30] and account for the lower limit of musical pitch [31].Then, a series of dense harmonic interval sieves was applied to each weighted ACF in order to quantify the neural activity at a given pitch period and its multiples [30,32]. Each sieve template (representing a single pitch) was composed of 500 μs wide bins situated at the fundamental pitch period (1/*f*_*0*_) and its integer multiples. All sieve templates with *f*_*0*_s between 25–1000 Hz (2 Hz steps) were used in the analysis. The salience for a given pitch was estimated by dividing the mean density of neural activity falling within the sieve bins by the mean density of activity in the whole distribution. ACF activity falling within sieve “windows” adds to the total pitch salience while information falling outside the “windows” reduces the total pitch salience [30]. By compounding the output of all sieves as a function of F0 we examine the relative strength of all possible pitches present in the FFR which may be associated with different perceived pitches. For each interval, we tracked the NPS magnitude at the F0 of the root note for each dyad. Previous work has shown that NPS is strongly modulated at the root F0 depending on the consonance/dissonance of multi-tone intervals and chords (Bidelman & Krishnan, 2009; Bidelman & Heinz, 2011).

Roughness estimation was performed on the recorded FFRs using the model described by Sethares [33] and later improved by Vassilakis [34, 35] to include the effects of register and waveform amplitude fluctuations described in the perceptual literature [1,36,37]. In this model, roughness is computed between any two sinusoids by considering both their frequency and amplitude relationship to one another. Consider frequencies *f*_*1*_, *f*_*2*_, with amplitudes *A*_*1*_, *A*_*2*_. We define *f*_min_ = min(*f*_1_, *f*_2_), *f*_max_ = max(*f*_1_, *f*_2_), *A*_min_ = min(*A*_1_, *A*_2_), and *A*_max_ = max(*A*_1_, *A*_2_). According to Vassilakis [38] p.141, the roughness (***R***) between these partials is given by

$$R=\;X^{0.1}\left(Y^{3.11}\right)Z$$ (1)

where ***X*** = *A*_min_*A_max,_ ***Y*** = 2*A*_min_/(*A*_min_+*A*_max_), $Z=\;e^{-b_{1}s(f_{max}-f_{min})}-\;e^{-b_{2}s(f_{max}-f_{min})}$, with parameters *b*_*1*_ = 3.5, *b*_*2*_ = 5.75, *s* = 0.24/(*s*_*1*_ *f*_min_+ *s*_*2*_), *s*_*1*_ = 0.0207, *s*_*2*_ = 18.96 chosen to fit empirical data on roughness and musical interval perception e.g.,[38,39–41]. The ***X*** term in Eq 1 represents the dependence of roughness on intensity (amplitude of the added sinusoids), the ***Y*** term, the dependence of roughness on the degree of amplitude fluctuation in the signal, and ***Z*,** the dependence of roughness/beating on the frequency separation of the two components [38]. Total roughness for a complex tone is then computed by summing the individual roughness from all possible (unique) pairs of harmonics in the signal. In order to apply this model to the recorded FFRs, the ten most prominent peaks were extracted from the response spectrum of each FFR and fed into a Matlab implementation of the model (see [35] for details).

### Behavioral judgments

After completing the electrophysiological recordings, participants rated intervals on a pleasantness scale from -4 (very unpleasant) to +4 (very unpleasant). To represent more faithfully the range of consonant and dissonant sounds used in behavioral experiments, we collected pleasantness ratings for eight different intervals ranging in semitone steps from the unison to the perfect fifth. Subjects were presented with ten repetitions of the eight dichotically presented intervals for the three different sound types. The 240 sounds were randomly interleaved in one block of testing. Responses were collected by means of button press.

## Results

Consistent with the literature, we found a correlation between NPS and behavioral responses for synthetic complex tones stimuli. For natural stimulus timbres however, a remarkably different pattern of responses was observed; NPS did not correlate with behavioral ratings of consonant and dissonant chords for naturalistic timbres (sax, voice). Interestingly, the NPS correlated with a simple measure of roughness applied to the FFR data.

**Fig 1:**
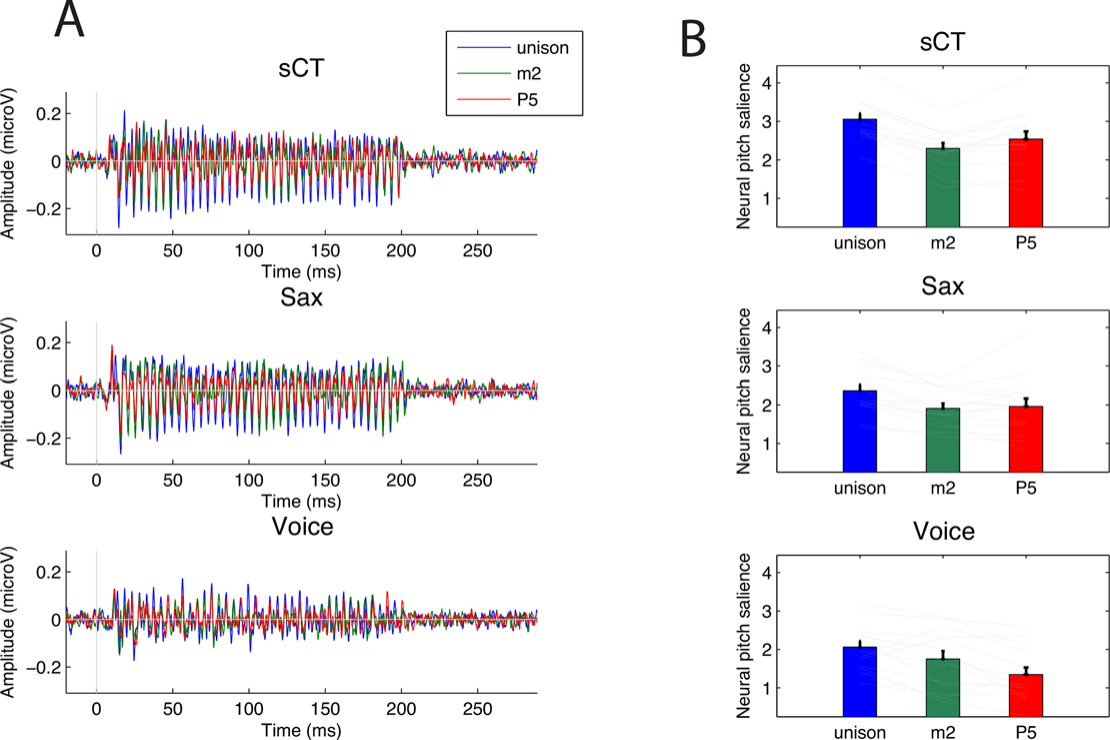
A) Mean Frequency Following Response (FFR) for the three interval types: unison (blue), minor second (m2, green) and perfect fifth (P5, blue). Top panel: synthetic complex tones; Middle panel: saxophone recordings; Bottom panel: voice recordings. B) Mean Neural pitch Salience (NPS, see methods) of the FFR for all types of intervals and stimulus types. Thin grey line show individual data and error bars represent +/- 1 SEM.

Fig 1A shows grand averaged FFR waveforms recorded for the three musical intervals and each sound types. Fig 1B shows the mean and individual NPS extracted from FFRs for the different stimulus condition. A mixed-model analysis of variance (ANOVA) was performed on the NPS data with sound type (sCT, sax, voice) and interval type (Unison, m2, P5) as within-subject factors. The ANOVA revealed significant main effects of sound type [F(2,26) = 18.83, p < 0.0001, effect size generalized-η^2^ = 0.27] and interval [F(2,26) = 30.40, p < 0.0001, η^2^ = 0.13]. More critically, we found a significant sound type x interval interaction [F(4,52) = 4.0, p = 0.006, η^2^ = 0.03], suggesting a difference in the pattern of responses obtained within each stimulus type, across the different musical intervals. Paired-sampled t-tests were used to compare the NPS obtained within each stimulus type between consonant and dissonant intervals. For sCT, the m2 NPS differed significantly from the NPS for the two other intervals (Unison/m2: p<0.0001; m2/P5: p = 0.03). For natural sounds, m2 NPS also differed significantly from the unison NPS (sax: p = 0.0002; voice: p = 0.04). However, difference between m2 and P5 was not significant for the saxophone (p = 0.66), and was significant for the voice (p = 0.01), but opposite of the expected direction (i.e., m2 > P5).

**Fig 2:**
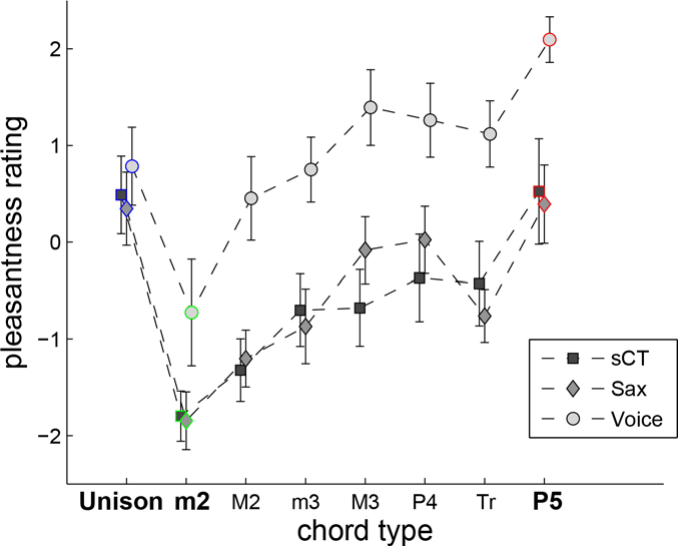
Mean pleasantness ratings for intervals ranging from the unison to the perfect fifth (P5) for synthetic complex tones (sCT, squares), saxophone recordings (Sax, diamonds) and voice recordings (Voice, circles). The three intervals for which FFR were recorded (unison, m2 and P5) are marked in blue, green and red respectively. Error bars represent +/- standard error about the mean.

Consistent with previous literature [4], behavioral ratings (Fig 2) demonstrated that consonant intervals were rated higher than dissonant intervals across all stimulus types. A repeated-measures ANOVA on the behavioral ratings revealed significant main effects of type [F(2,26) = 8.13, p < 0.002, η^2^ = 0.17] and interval [F(7,91) = 14.12, p < 0.0001, η^2^ = 0.20], as well as a significant interaction between the two factors [F(14,182) = 1.77, p < 0.046, η^2^ = 0.02]. Paired-sampled t-tests were used to compare the ratings obtained for the consonant and dissonant intervals used in the EEG experiment. These analyses revealed that within each stimulus type, m2 ratings were significantly lower than unison ratings (sCT: p = 0.0005; sax: p = 0.0003; voice: p = 0.13) and P5 ratings (sCT: p = 0.001; sax: p = 0.0002; voice: p<0.0001).

**Fig 3:**
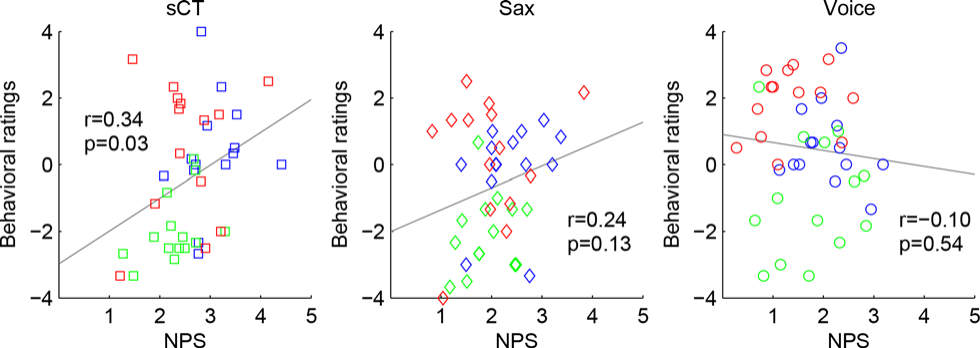
Pleasantness ratings plotted against Neural Pitch Salience (NPS) for synthetic complex tones (sCT, left panel), saxophone recordings (Sax, middle panel) and voice recordings (Voice, right panel). The three intervals are color-coded: Unison: blue; m2: green; P5: red. Correlation coefficient and p value for the correlation are reported in each panel.

Fig 3 shows each individual’s behavioral ratings plotted against their FFR NPS for each type of interval. For sCT, pleasantness ratings strongly correlated with the neural pitch salience (Fig 3) [r = 0.34, p = 0.03], consistent with the notion that stronger neural harmonicity is related to consonance percepts [8]. For the natural sounds, however, behavioral ratings were not correlated with neural measures for either the Sax (r = 0.24, p = 0.13) nor Voice (r = -0.10, p = 0.54) timbres.

**Fig 4:**
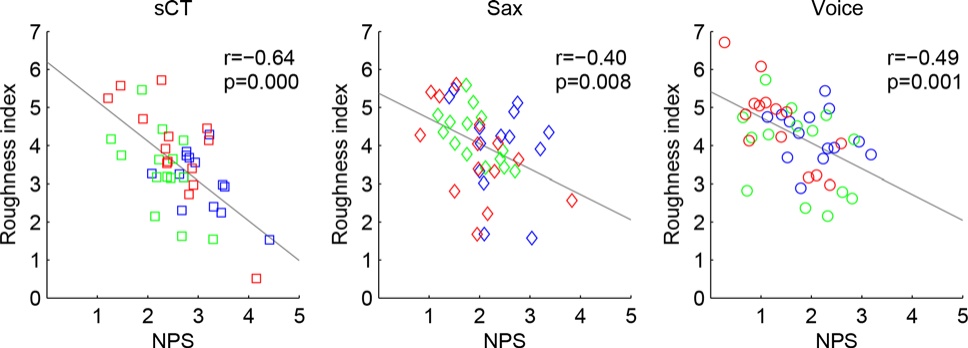
Roughness index of the FFRs plotted against Neural Pitch Salience (NPS) for complex tones (sCT, left panel), saxophone recordings (Sax, middle panel) and voice recordings (Voice, right panel). The different intervals are color-coded: Unison: blue; m2: green; P5: red. Correlation coefficient and p value for the correlation are reported in each panel.

Finally, Fig 4 shows the NPS plotted against an estimate of roughness based on the Vassilakis model [35] calculated on the individual FFRs. We observed strong negative correlations between NPS and roughness both when all types of sounds were considered [r = -0.57, p<0.00001] and for each type of sound individually [sCT: r = -0.64, p<0.00001; Sax: r = -0.40, p = 0.0079; Voice: r = -0.49, p = 0.0009]. To ensure that these correlations were not driven by between-subjects variability we computed correlation coefficients for each individual participant (between NPS and behavioral data obtained for the three sound types x three intervals). A one-sample t-test revealed that correlation coefficients across the sample of 14 participants were significantly different from 0 (p = 0.001).

## Discussion

We recorded the brainstem frequency-following response (FFR) to consonant and dissonant dyads (unison, m2 and P5) for synthetic and natural sounds. We replicated the documented correlation between NPS (a measure of neural harmonicity of the FFR) and behavioral ratings for synthetic complex tones whereby larger NPS was associated with consonant intervals rated as more pleasant [8,18,42]. However, using intervals composed of other instrumental timbres, we failed to observe this brain-behavioral correlation for natural sounds. Importantly however, we believe that this absence of correlation cannot be attributed to the poorer SNR obtained in FFR for natural sounds that would have swamped out differences between NPS for the different dyads. Indeed, significant differences were observed between NPS for m2 and P5 in the case of natural voice stimuli, but this effect was in the direction opposite to the one found with synthetic stimuli, with the dissonant m2 resulting in a larger NPS than the consonant P5.

The fact that NPS does correlate with behavior only in the case of synthetic complex tones suggests that it cannot be considered a *universal* neural correlate of consonance and dissonance perception. Behavioral ratings are consistent regardless of the stimulus timbre; synthetic and natural chords are rated similarly even by musically naïve listeners [4] and this result should be mirrored by a neural correlate of consonance perception.

### Synthetic complex tones are not optimal to study consonance

Because synthetic complex tones are conveniently easy to generate, control and manipulate, they have been extensively used in the behavioral literature on consonance and dissonance perception [1–3,33,43]. These complex tones are harmonic and thus share the harmonic frequency relationships of natural musical stimuli, but differ in a number of other ways (amplitude and phase of the partials, attack cues, etc.) and do not capture the variability found in natural music listening situations. Synthetic stimuli have been advantageous for recording brainstem responses as these stimuli are optimal for evoking the FFR compared to pure tones or other naturalistic sounds [44]. However, it was recently shown that whereas roughness correlates with behavioral ratings for such tones, it is not the case for all natural sounds [7]. This specificity of synthetic complex tones has been decisive in the prevalent role that beating theories of dissonance have played over the past decades.

### Dichotic presentation, NPS and roughness

There is now some consensus that harmonicity rather than roughness is the acoustic correlate of consonance perception. To circumvent roughness, it has become customary to use dichotic presentation of chords [8,17–19,24], in order to prevent the generation of cochlear distortion products [45] that produce roughness [33]. The idea is that the contribution of roughness will be eliminated by this dichotic presentation because the two sounds do not pass through the same auditory filters, largely eliminating roughness cues but retaining the true acoustic correlate of consonance, i.e., harmonicity.

In an attempt to understand where the correlation between behavior and NPS observed for sCT could come from, we applied the Vassilakis [35] model of roughness to the neural data. This analysis was motivated by the fact that, like NPS, the amount of roughness present in the stimuli correlates with behavioral ratings only for sCTs [7]. This FFR roughness correlated significantly with NPS for all types of sounds. It is not possible to know which proportion of this “roughness” is reintroduced by the combination of sounds at the level of the brainstem [46], despite the dichotic presentation—because the tonotopic organization of the inferior colliculus (a nuclei of the brainstem where dichotic inputs converge) is similar to critical bands of the cochlea [47]–and which proportion is due to the summing of neural data coming from different populations performed when recording the FFR. Nevertheless, the significant correlation observed between roughness and NPS applied to the FFR reveals that the two metrics capture a similar aspect of the input FFR. This result thus reveals that NPS is not, as originally thought, an index of harmonicity not contaminated by roughness. It also explains why NPS correlates with behavior for sCT and not for other types of sounds.

### NPS, a brainstem correlate of consonance perception?

NPS, by construction, does reflect a form of harmonicity due to the pitch sieve analysis, but the way it is implemented here cannot account for general consonance perception. From a theoretical point of view, if we imagine that the brainstem’s FFR represents faithfully the spectral content of sounds, the artificially rich lower part of the spectrum of synthetic tones will necessarily result in emphasized differences in the NPS between consonant and dissonant chords. Indeed, energy inside the harmonic series of the F0 will boost the NPS, whereas energy outside this harmonic series will reduce it. The first five harmonics of the upper tone of the minor second, for example here, will thus strongly reduce the sieve analysis implemented in the NPS, resulting in a large contrast between NPS for m2 and unison. The fact that NPS for saxophone sounds (that have a rather rich spectrum) resemble more NPS for sCT than NPS for voice sounds (which amplitude of spectrum falls out rapidly after the first harmonic) is consistent with this idea.

Consequently, the present results might be an indication that, even if acoustic correlates that are necessary to evoke percepts of consonance and dissonance are present at the level of the brainstem, the percept itself might emerge at a higher level (e.g. cortical, see [24,48]) where the neural signal has been modified by the numerous sites of nonlinearity found at each stages of the auditory pathway (see e.g [49]). As was argued by Gockel et al. [50] for pitch, we believe that the FFR may reflect consonance-bearing information but is not a direct representation of consonance. To further investigate the hypothesis that consonance arises only at the level of the cortex, one possibility would be to repeat this experiment (timbral variations on intervals) but this time looking at cortical ERPs. If consonance arises at higher levels (e.g., A1) then responses should not only link to behavior but should also be invariant across timbres.

## Conclusions

In the present experiment, we have shown that a robust auditory brainstem response can be recorded in response to natural musical sounds. Neural Pitch Salience (i.e., harmonicity) computed on neural responses correlated with roughness but not with behavioral ratings, suggesting that results obtained with synthetic tones (earlier and in this experiment) are a stimulus-driven phenomenon. Therefore the NPS cannot be considered a general correlate of consonance perception. Unfortunately, we are not able to provide an alternative correlate of consonance perception in the brainstem. However, we felt it was important to communicate these results, considering the growing number of studies using variants of NPS to study brainstem correlates of consonance. In the light of these results, caution is suggested regarding the exclusive use of synthetic stimuli in future experiments, as they represent a special case and generalizability to other stimuli, particularly natural ones, is not warranted.
